# An Energy-Efficient Cluster-Based Vehicle Detection on Road Network Using Intention Numeration Method

**DOI:** 10.1155/2015/613923

**Published:** 2015-02-22

**Authors:** Deepa Devasenapathy, Kathiravan Kannan

**Affiliations:** ^1^Department of Information Technology, Easwari Engineering College, Chennai 600089, India; ^2^Department of Computer Science and Engineering, Easwari Engineering College, Chennai 600089, India

## Abstract

The traffic in the road network is progressively increasing at a greater extent. Good knowledge of network traffic can minimize congestions using information pertaining to road network obtained with the aid of communal callers, pavement detectors, and so on. Using these methods, low featured information is generated with respect to the user in the road network. Although the existing schemes obtain urban traffic information, they fail to calculate the energy drain rate of nodes and to locate equilibrium between the overhead and quality of the routing protocol that renders a great challenge. Thus, an energy-efficient cluster-based vehicle detection in road network using the intention numeration method (CVDRN-IN) is developed. Initially, sensor nodes that detect a vehicle are grouped into separate clusters. Further, we approximate the strength of the node drain rate for a cluster using polynomial regression function. In addition, the total node energy is estimated by taking the integral over the area. Finally, enhanced data aggregation is performed to reduce the amount of data transmission using digital signature tree. The experimental performance is evaluated with Dodgers loop sensor data set from UCI repository and the performance evaluation outperforms existing work on energy consumption, clustering efficiency, and node drain rate.

## 1. Introduction

WSN is emerging as an increasingly used application that might competently examine, monitor, and control physical world via a huge number of small, low-priced sensor nodes. Most of the existing research on movement monitoring focuses on dealing with a single vehicle, where vehicle numeration is not needed.

The positioning and the location of numerous vehicles provide tracking of vehicles. For more data congregation with the least amount of restricted resources, sensor nodes should be processed to significantly collect the data by constructing restricted choices [1]. But the real deployment of wireless networks remains unaddressed.

Several research areas on data aggregation focused on homomorphic encryption mechanisms to hide communication during aggregation. However, the homomorphic schemes were not satisfied for multiapplication environments. Therefore, in [2], a new concealed data aggregation scheme was presented in wireless sensor network. The performance evaluation showed that CDAMA was highly applicable on WSNs and the number of groups or applications cannot be extended to an overall large capacity and compromised the computation cost.

Certain applications including stock exchange, portfolio maintenance for financial decision making, and monitoring sensor nodes make extensive use of dynamic data. For applications involving dynamic data, the data obtained from one or more sources are aggregated to obtain the result. At the same time a low-cost, scalable method was presented in [3] that included dynamic items for continuous aggregation. But a promising solution to be obtained was the multiple invocations considering the hierarchy of data aggregators was unaddressed. A novel data-delivery scheme for delay-sensitive traffic was designed in [4] to minimize the consumption of energy in wireless networks but wait time was not measured. The dynamic motion model as designed in [5] proposed segmentation using edge based dilation to segment only a specific area for moving object rather than the entire area resulting in robustness.

As WSNs are deployed in hostile environments, sensitive information has to be transmitted. The paper presented in [6] identified the relationship between integrity and data aggregation involved in wireless sensor networks.

A possible approach to securitize the routing procedure using existing traffic segmentation also called DRINA [7] utilized processing capability offered by the intermediary sensor nodes all along the direction-finding paths. Aggregation points using DRINA or one of the possible existing traffic segmentation methods that provided an efficient mechanism for fault tolerance resulting in the improved delivery rate. Though the number of messages for setting the routing tree was reduced tradeoff was achieved between the overhead and quality of the routing tree.

In the context of improving the energy efficiency in a road network, intention numeration should provide certain characteristics like reducing the energy consumption, reducing overhead by increasing packet delivery ratio, and improving the clustering efficiency by designing cluster-based vehicle detection with minimum node drain rate. In this paper, we solve the problem of cluster efficiency and how to minimize energy consumption using the intention numeration method. We proposed a cluster-based vehicle detection in road network using intention numeration method which emphasizes sensor nodes that detect vehicle and is grouped according to a node energy adaptive clustering. Compared to the existing vehicle detection, CVDRN-IN uses polynomial regression function to obtain the strength of the node drain rate. We also construct a digital signature tree using enhanced data aggregation to reduce the amount of data transmitted. Extensive performance evaluated using the Dodgers loop sensor data set provides better results on energy consumption, clustering efficiency, and node drain rate.

The major contributions of this paper are as follows:

- to design node energy adaptive clustering, to detect a vehicle and form separate clusters accordingly;
- to reduce the amount of data transmission using digital signature tree;
- to present cluster-based vehicle detection in road traffic network using the intention numeration method.

The rest of the paper is organized as follows.  Section 2 presents a brief outline of the state-of-the-art methods involved in road traffic.  Section 3 presents the proposed methodology.  Section 4 provides experiments and discussions on CVDRN-IN. Finally, Section 5 includes the concluding remarks.

## 2. Related Work

One of the key technologies involved in the intelligent transportation systems is the identification of a moving vehicle. Traffic surveillance is now being more significant. The work in [8] presented an intelligent vehicle counting scheme supported with the blob examination for traffic surveillance. The author in [9] proposed an empty parking slot identification and tracking scheme to identify the sensors of an around view monitor (AVM) scheme and an ultrasonic sensor-supported parking scheme.

Moving vehicle identification is a significant part of the traffic surveillance scheme. The author in [10] provided a fast and robust background algorithm supported with unified spatiotemporal background and foreground representation. The association between the adjacent pixels was processed using high levels of identification accuracy of the active background scene.

The author in [11] presented an efficient signal processing method to identify the moving vehicles for cross range systems using a passive bistatic radar (PBR) supported with signals emitted by a WiFi router. The author in [12] analyzed different multichannel techniques involving spaceborne multichannel synthetic aperture radar for ground tracking.

In traffic intelligent transportation system, classification of vehicles acts as the most demanding task to obtain the traffic vehicles movement. In [13], a novel identification scheme was presented via numerous time-spatial images (TSIs), from which every image was processed from a recorded frame of a video. Such multiple TSIs provide less chance of identification of unauthorized entries in the road network. A specific VSN architecture was designed in [14] that integrated nonvaluable individual sensed data and obtained the road scenarios by applying a protocol.

Complex moving vehicle detection is one of the significant means of eradicating the occurrence of accidents. In [15], a traffic surveillance scheme, called the active visual representation (DVM), was presented by detecting complex movement of vehicles on a highway. In [16], the author proceeded with the dispersed identification of moving vehicles on roads and highways. The dangerous vehicle detection protocol (DVDP) was processed with the detection of drivers who examine the assigned speed limit.

In-network data aggregation is a highly significant communication mechanism to minimize the bandwidth requirements in vehicular ad hoc networks (VANETs). The work in [17] portrayed the requirements and introduced modeling approach and facilitated joint optimization.

During the design considerations of WSN, compromised sensor nodes give data falsification during both data aggregation and data forwarding. Certain researchers consider false data injections during data forwarding. However, in [18] a protocol concerning data aggregation and authentication was provided to integrate data falsification with data aggregation and confidentiality. However, those clustering methods proved to be effective only in certain type of sensing scenario. The work presented in [19] selected suitable clustering technique using network status, by increasing the efficiency of data aggregation and consumption of energy.

For vehicle categorization, analysis of presentation of three common categorizations based on two processing methods with the empirical data was presented in [20]. In [2], an intelligent sensor network was processed for the detection, identification, and categorization of moving vehicle. Wireless sensors are processed with the preliminary objective to identify the coordinates of moving vehicles. But integrity was not applied when road network was occupied by a large number of vehicles. To provide a solution to the aforementioned problems, in this paper, we propose a cluster-based vehicle detection using the intention numeration method to minimize the energy. In the forthcoming section, a detailed design of the proposed work and the advantage of using it with the existing state-of-the-art methods are presented.

## 3. Design of CVDRN-IN

In this section a cluster-based vehicle detection in road network using enhanced data aggregation with intention numeration method is presented. Then a system model is designed using enhanced data aggregation with intention numeration followed by the three phases involved in CVDRN-IN.

### 3.1. Enhanced Data Aggregation with Intention Numeration Method

Sensors in WSN can be organized to sense the information flow, rapidity, and occupancy by examining the signal around them. In road network, fully charged battery powered sensor nodes are arbitrarily placed in an area with a fixed source and sink nodes. An intention numeration method based on node energy (IN-node energy) distinguishes between power computation, elevated accuracy, and node energy capacity requirement.

Intention numeration is to count the number of intentions (i.e., the destination nodes or the intended nodes that are involved in the vehicle detection) nodes in wireless sensor network with dimension equal to n where the sensor nodes are placed in a random manner. Each intended node is considered to be a point source of signal, for example, power computation, elevated accuracy, and node energy capacity, whose strength (or amplitude) attenuates with all queue's distance.

**Figure 1:**
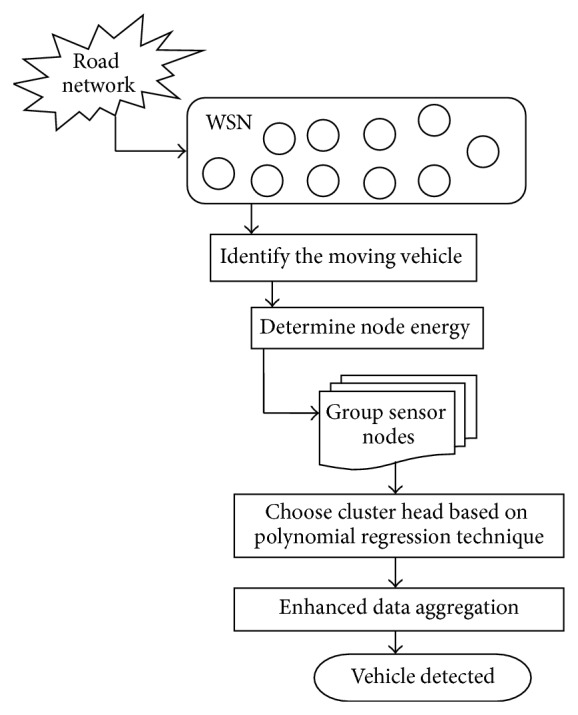
Architecture diagram of CVDRN-IN.

The architecture diagram of the proposed cluster-based vehicle detection in road network is shown in Figure 1.

The process of enhanced data aggregation with the intention numeration method for detecting the movement of the vehicle for a high traffic road network is illustrated in Figure 1. By determining the movement of vehicles in the network, the necessary information is extracted from the network. The proposed enhanced data aggregation with intention numeration method is processed under three phases.

The first phase in CVDRN-IN clusters the sensor nodes based on vehicle identification. The second phase chooses the cluster head by implementing polynomial regression technique. The third and final phase performs enhanced data aggregation using Merkle hash tree by estimating the size of the network and reduces the large amount of transmission.

### 3.2. System Model

In this section, the system model is presented by including certain notations and notions. Vehicles turn up to the traffic light junction (TLI) consistent with the definite arbitrary allocation and disappear after some time. For easy allocation, let us assume that each region of the TLI is represented as M/M/1 queue. All queues' distance end to end for all dynamic directions is set to zero. The distances among any pair of the junctions are determined and are equal to a predefined base distance (*d*). The vehicle recognition system for CVDRN-IN includes four sets as follows:

1. sensor to notice the vehicle's signals produced by vehicles,
2. processor to progress the obtained data from sensor nodes,
3. communication unit to transport the user obtained data to the BS for processing,
4. energy source.

Let us assume that there are n sets of sensors *S*_1_, *S*_2_,…, *S*_*n*_ on road *R* and at a particular time interval *n* time series are observed:

(1) $$\begin{array}{c} \left\{{\text{fl}}_{t\left(h\right)}^{k},{\text{fl}}_{t\left(h+1\right)}^{k},\ldots ,{\text{fl}}_{t\left(j\right)}^{k}\right\}, \end{array}$$

where fl indicates a definite type of traffic constraint (e.g., flow, occupancy, speed) and *k* denotes the sensor ID. The road network condition is characterized by time series to identify the configuration of the state of the road network. Based on the traffic junction points, the system operation is split into various time slots in which each traffic junction point operates. Then the collected traffic information combined by the BS is passed onto the road network for traffic signals in an active fashion along with the vehicles in each traffic signal. The forthcoming section discusses in detail the three phases involved in CVDRN-IN.

### 3.3. Adaptive Node Energy Clustering

The first phase in CVDRN-IN is to cluster the sensor nodes in the network area based on the identification of vehicles. Once the moving vehicles are identified, the energy drain rate is observed. In a similar manner, the node energy drain rate is calculated for the entire cluster and the number of vehicles detected is classified.

Let us consider a set of sensor nodes *S* = {*S*_1_,…, *S*_*n*_} and assume that *L* = 〈*l*_1_, *l*_2_,…, *l*_*m*_〉 is an association path of any vehicle with time-span equal to *m*. Here *l*_*i*_ signifies the position of a sensor node that was called consecutively by the vehicle based on its incoming and outgoing processes. Let *D* = 〈*D*_1_, *D*_2_,…, *D*_*n*_〉 be the movement of vehicles with dimension equal to *n*, produced by different vehicles.

The next step is to calculate the subsequent location of the moving vehicle and keep viewing the moving vehicles. Since the sensor nodes reveal the uniqueness of clustering process, the clustering mechanism is utilized to figure out the hierarchical formation of the sensor networks. The route path of a moving vehicle VO is formed by specifying the start_point (*s*), that is, the starting time, and the end_point (*e*), that is, ending time. The movement of vehicles for a specified route path, that is, from *s* to *e*, is denoted by

(2) $$\begin{array}{c} M\left(\text{VO}\right)s⟶e=\frac{\text{VO}\left(R,s\right)}{\text{VO}\left(R,s\right)\text{VO}\left(e,r\right)}, \end{array}$$

where (VO) denotes the vehicle under consideration *R* measured from the starting point *s* to the ending point *e* in a traffic road network.

Sensors in conventional wireless sensor networks are dispersed all along the roadway so that each sensor node monitors definite number of predetermined locations. In CVDRN-IN, let us assume that at a specified time interval *t* only one vehicle is at a definite position with a single route path to be followed. During this case, data collection initiates when movement of the vehicle is detected. So, from the primary point of progression of vehicles in road path, a sequence of sensor evaluations is composed at a predetermined time from one another. The locations of each individual vehicle inside the sensor communicative range are then accumulated for each reading. Each moving vehicle is assigned with a unique identifier and the linear amount of the moving vehicle and its intermediate points are stored and passed to the nearby base station. The crucial measures of two or more moving vehicles on a road provide the support that differentiates set of vital motion associations of the vehicles.

**Figure 2:**
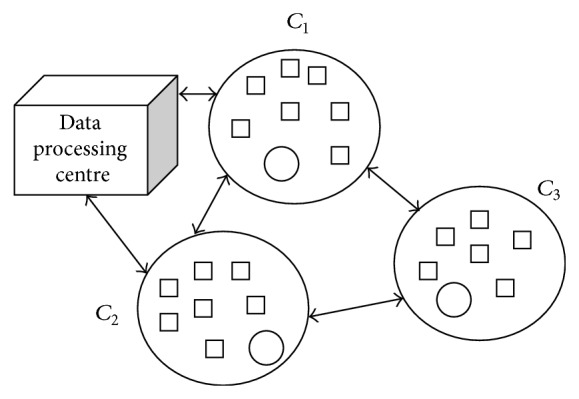
Adaptive node energy clustering.

Once the set of information regarding the movement of vehicles is collected from the route path, the sensor nodes are grouped. The process of grouping the sensors is shown in Figure 2.

After the formation of clusters, the data collected of the number of vehicles are evaluated by each set of clusters in the road network and determine the sensor node energy required to process all those collected data simultaneously.

Algorithm 1 describes the process of adaptive node energy clustering.

Initially, the number of vehicles under consideration is observed in the road network *R*. Then, for each vehicle in the network, the movement of vehicles for a specified route path is measured from the starting point “*s*” to the ending point “*e*.” The nodes are then clustered in the particular area which collects the information of the vehicles “VO.” Finally, the clusters are separated as *C*_1_, *C*_2_,…, *C*_*n*_. By applying the above algorithm, the process of clustering is performed with appropriate set of sensor nodes in the road network. Once the clusters are formed, the next step is to determine the cluster head. The forthcoming section discusses in detail the formation of cluster head using polynomial regression function.

### 3.4. Formation of Cluster Head Using Polynomial Regression Function

The second phase involved in CVDRN-IN is choosing the cluster head by using polynomial regression technique. The formation of cluster head is performed to approximate the node drain rate strength over a cluster and the total node energy capacity is estimated by taking the integral of the function over the area.

For each cluster in the road network, the node drain rate is estimated based on the sensor nodes holding the vehicle information. For each cluster, the node drain rate is estimated as

(3) $$\begin{array}{c} \text{DR}\left(C_{n}\right)=F_{i}\left(E\right)-\frac{C_{i}\left(E\right)}{\text{Cur}T}-\text{Pre}T, \end{array}$$

where DR(*C*_*n*_) represents the node drain rate of the *n*th cluster with *F*_*i*_(*E*) denoting the *i*th node energy level of the former state and *C*_*i*_(*E*) denoting the *i*th node energy level of the present state. In a similar manner, the time interval is represented as Cur*T* and Pre*T* being the current and the previous time interval, respectively.

Using (2), the node drain rate for each cluster is estimated. Once the node drain rate for each cluster is estimated, the node which has the least DR (drain rate) is chosen as the cluster head, which is used as an approximation for the node drain rate strength over a cluster.

If several nodes in the cluster have the same number of node drain rate strengths, then it is obviously difficult to select the cluster head for better movement. So, polynomial models are utilized to approximate the node drain level of each node in the cluster and choose the cluster head accordingly.

Consider a set of sensor nodes *S* = {*S*_1_, *S*_2_,…, *S*_*n*_} with the vehicle information as VO = {VO_1_, VO_2_,…, VO_*n*_}. The basic assumption to be followed using polynomial regression in CVDRN-IN is that a data set of *n* paired (S, VO) pairs is expressed as

(4) $$\begin{array}{c} \left(S_{1},{\text{VO}}_{1}\right),\left(S_{2},{\text{VO}}_{2}\right),\left(S_{3},{\text{VO}}_{3}\right),\ldots ,\left(S_{n},{\text{VO}}_{n}\right). \end{array}$$

The equation in (4) is measured using a least-squares technique to generate an analytical polynomial equation of degree *p* to decrease the remaining *R* at every data point expressed in

(5) $$\begin{array}{c} C=C_{0}+C_{1}E+C_{2}E^{2}+C_{3}E^{3}+\cdots +C_{p}E^{p},\;p<n,\\ R_{i}=C_{0}-C_{1}E-C_{2}E^{2}-C_{3}E^{3}-\cdots -C_{p}E^{p}. \end{array}$$

The polynomial regression representation for selecting the cluster head is expressed in terms of matrix as follows:

(6) C1C2⋮Cn=1VO11VO22VO23⋯VOn11VO12VO22VO22⋯VOn2⋮⋮⋮⋮⋯⋮1VO1nVO2nVO3n⋯VOmn·s1s2⋮sn,

where the vehicles' superscript value defines measuring of vehicle in particular path *n* inside the cluster *C*_*n*_. With this matrix representation, the vehicle is assigned with appropriate sensor nodes in appropriate cluster. For each cluster, the node draining rate is evaluated and processed with the area of the cluster size. Once the cluster head is determined, the total energy capacity *E*(*C*_*n*_) is evaluated based on the area of the cluster Area(*C*_*n*_) in the road network:

(7) $$\begin{array}{c} \text{Area}\left(C_{n}\right)=\frac{\text{No}.\;\text{of}\;\text{sensor}\;\text{nodes}\;\text{in}\;C}{D}*E\left(C_{n}\right), \end{array}$$

where *D* denotes the cluster *C*. By identifying the area for the cluster Area(*C*_*n*_), the total energy capacity is evaluated as

(8) $$\begin{array}{c} \text{Tot}E\left(C_{n}\right)\;=\;\int \text{Area}(C_{n}). \end{array}$$

With these values, the energy capacity is determined and processed based on the number of sensor nodes on the road network and determines the vehicle movement categories.

### 3.5. Digital Signature-Based Enhanced Data Aggregation

The third phase in CVDRN-IN is performing enhanced data aggregation using digital signature. The digital signature-based enhanced data aggregation (DSEDA) model consists of three parts, namely, key generation, enhanced data aggregation, and confirmation.

The first part involved in digital signature-based enhanced data aggregation is the key generation that generates a private key for each cluster, and each sensor node belonging to the specific cluster has a share of the private key. Each sensor node senses the movement of the vehicle (MV) and then encrypts it using its share of the cluster's private key.

Let us assume that *K*_*P*_ denotes the private key shared between the sensor node *S* and the base station. The encryption of the sensed movement of vehicle MV based on sensor node *P* is evaluated as follows:

(9) $$\begin{array}{c} E_{K_{P}}=\left({\text{MV}}_{P}+K_{P}\right). \end{array}$$

The second part involved in DSEDA ensures that base station does not accept invalid aggregation results from the cluster head. The digital signature-based enhanced data aggregation is performed on the encryption of the sensed movement of vehicle MV received from the corresponding sensor node (node *P* and node *Q*) which is given as follows:

(10) Digital  Signature  Enhanced  Data  Aggregation(DSEDA)=EKP+EKQ+EKQR.

Based on the movement of the vehicle, the sensor node sends its ID, data packet, and two message authentication codes. This message authentication code includes the sensor node ID, with the vehicle information and the message authentication code MAC_ID_ (S_ID_, VO_1_). The first key is shared with the aggregator and the second key is shared with the base station. The sensor node sends these data to the cluster head, which aggregates the nodes MVs and measures their average.

The cluster head then broadcasts the nodes based on the movement of the vehicle to the specified sensor nodes belonging to a cluster. It then evaluates their MVs with the measure of average. If the result of the measure of average is less than a constant factor, the sensor node generates a digital signature on the measure of average using its share of the cluster's private key and then sends it to the cluster head. The cluster head then combines these digital signatures into a full-fledged signature and sends it in addition to the measure of the average value to the base station.

The third part involved in DSEDA is the verification phase where the base station interacts with sensor nodes and aggregators in order to verify the aggregation results. When the results of digital signature-based data aggregation reach the base station, the base station exposes the symmetric keys shared with every node. Every cluster head now verifies whether the packet data and message authentication code stored in its sensor node are matched or not. If the cluster head detects the message authentication code to be inconsistent with that of its sensor node, it sends out an alarm signal to the base station along with the message authentication code evaluated using the sensor node's temporary key.

With these values, the energy capacity is determined and processed based on the number of sensor nodes in the road network. Based on these values, the energy capacity is identified and determines the vehicle movement categories.

## 4. Performance Evaluation

In this section, an experimental evaluation is done to estimate the performance of the proposed cluster-based vehicle detection in road network using enhanced data aggregation with intention numeration method. The experimental evaluation is done with the Dodgers loop sensor data set extracted from the UCI repository. These loop sensor measurements were received from the Freeway Performance Measurement System (PeMS), http://its.berkeley.edu/publications/UCB/2007/CWP/UCB-ITS-CWP-2007-7.pdf. The characteristics of the data set and attributes in Dodgers loop sensor data set are multivariate and integer categorical. The total number of instances is 50400 and the number of attributes is 3.

These Dodgers loop sensor data were composed for Glendale on a slope for the 101 North Freeway in Los Angeles.  Table 1 lists the attributes considered in Dodgers loop sensor data that includes attribute information and event files. The attribute information used is the date, time, and count that denotes the military data and time in addition to the number of cars measured. The event information includes data, beginning event time and ending event time, team playing, and win/loss situation. It is sufficient to perceive abnormal traffic after Dodgers game and deeply utilized by game traffic so that the indicator for the further traffic is excessively apparent. With this data set, an experimental evaluation is carried out to estimate the best approach for the vehicle identification scheme.

The performance of the proposed cluster-based vehicle detection CVDRN-IN in road network using enhanced data aggregation with intention numeration methodis compared with the existing adaptive traffic segmentation [7] method which is illustrated in this section (Section 5). The graph describes the comparative evaluation of the proposed IN based scheme with adaptive traffic segmentation method.

**Figure 3:**
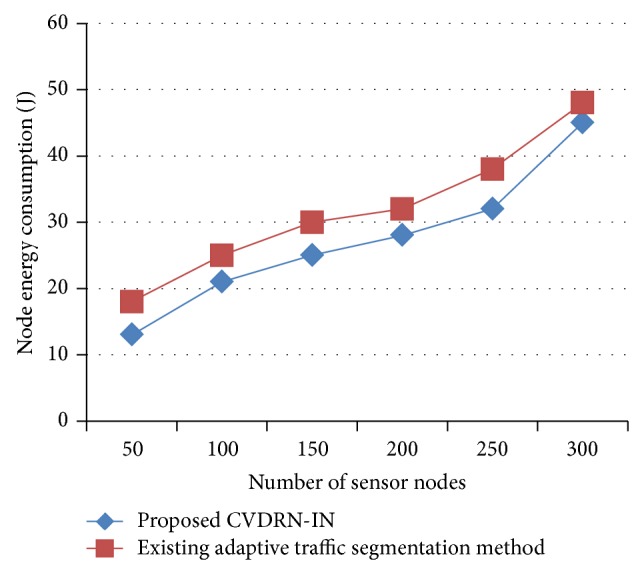
Measure of node energy consumption.

The node energy consumption is measured based on the number of sensor nodes or the number of cars measured in the previous five minutes using Dodgers loop sensor data in the network area. The value of the proposed CVDRN-IN is compared with the existing adaptive traffic segmentation [7] method.  Figure 3 describes the process of consumption of energy required to process the specified sensor nodes in terms of Joules in the road network with the number of sensor nodes in the range of 50 to 300 with the beginning event time as 14:00 and ending event time as 19:00. Compared to the existing adaptive traffic segmentation [7] method, the CVDRN-IN consumes less energy even when more numbers of sensor nodes are present. This is because the proposed CVDRN-IN method clusters the sensor nodes based on the movement of vehicles. Since adaptive node energy clustering is performed, energy consumption for movement of vehicles is noted appreciably whereas in the existing method the energy conservation mechanism was provided but at the cost of increased routing protocol. The improvement of energy consumption is significantly improved by 3–8% compared to the existing adaptive traffic segmentation [7] method.

**Figure 4:**
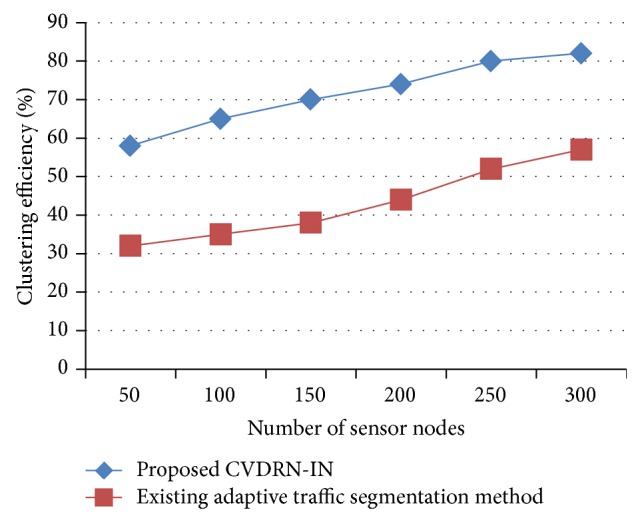
Measure of clustering efficiency.

Figure 4 describes the efficiency of cluster measured based on the number of sensor nodes in the network area. The clustering efficiency (in terms of %) is measured based on the rate at which the sensor nodes are grouped for collecting the information of vehicle movement and obtained through the value of *W*/*L* score obtained through the Dodger data set. Based on each ratio of *W*/*L* score, the clustering efficiency also gets improved. Compared to the existing adaptive traffic segmentation [7] method, the proposed CVDRN-IN has a higher cluster efficiency. This is because the clustering is performed based on the node drain rate of each of the sensor nodes in the network, whereas, in the existing adaptive traffic segmentation, efficiency was improved in terms of packets being processed. Though the packet processing efficiency was improved, repeated similar packets were processed, resulting in increased computation overhead. The variance in the clustering efficiency is 20–25% higher in the proposed CVDRN-IN than in the existing adaptive traffic segmentation [7] method.

**Figure 5:**
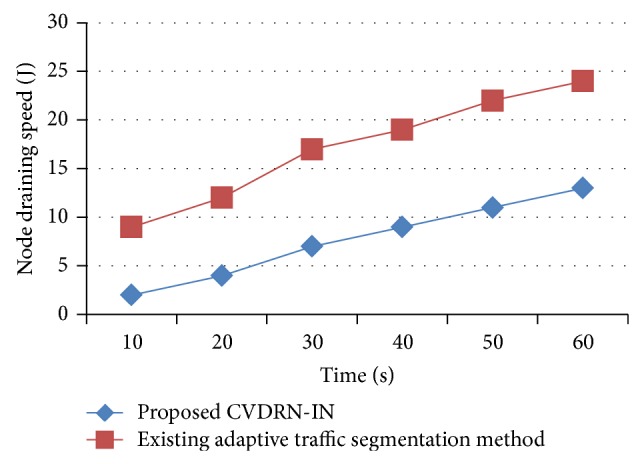
Measure of node draining speed.

Figure 5 describes the measurement of node draining speed in terms of Joules based on the time taken to place the vehicles in the road network. The beginning event time and ending event time for one cycle were considered between 13:00 and 16:00, 19:00 and 21:00, respectively. In this way the observations were made with an interval of 10-second gap. Compared to the existing adaptive traffic segmentation [7] method, the proposed CVDRN-IN method provides less draining speed. The less draining speed observed in CVDRN-IN is due to the fact that the sensor nodes in the network are fixed and it detects only the movement of vehicles on the road network. Normally, when the sensor nodes are fixed, the draining speed of the node is very low compared to the dynamic sensor nodes of existing methods and improved by 10–30% compared to the existing adaptive traffic segmentation [7].

**Figure 6:**
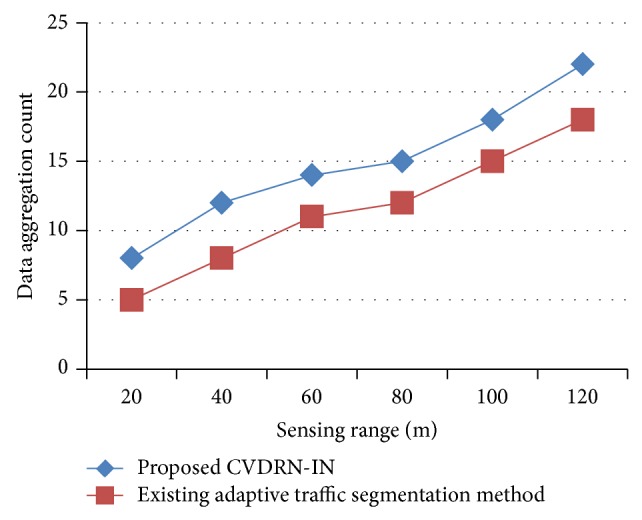
Measure of data aggregation count.

Figure 6 illustrates the data aggregation count offered by CVDRN-IN and comparison analysis is made with the existing adaptive traffic segmentation [7] with the sensing range of 20 m to 120 m observed in the network with the beginning event time as 14:00 and ending event time as 17:00. From the figure it is illustrative that the level of data aggregation count using cluster-based vehicle detection in road traffic network maintains the highest aggregation efficiency. This is because the movement of the vehicle is grouped into separate clusters using intention numeration methods based on node energy, whereas the existing method was based on the packet transmission rate. The aggregation efficiency achieved using CVDRN-IN is 3–5% higher than the existing adaptive traffic segmentation [7].

**Figure 7:**
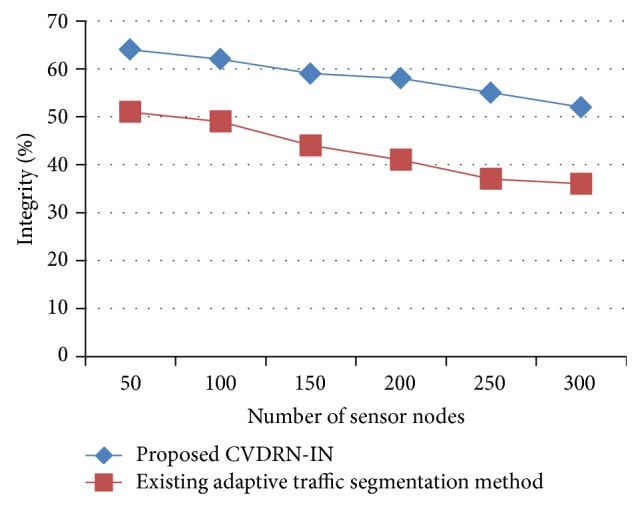
Measure of integrity.

Figure 7 illustrates the measure of integrity provided using CVDRN-IN and comparison made with existing adaptive traffic segmentation [7] method. In the above analysis, the integrity measured in terms of % offered using CVDRN-IN is significantly large. To argue with this point, the performance gain in terms of integrity is measured for the count value or number of sensor nodes ranging from 50 to 300 for different beginning event and ending event time. The integrity is high in CVDRN-IN because the sensor node generates a digital signature on the measure of average using its share of the cluster's private key and then sends it to the cluster head. The variance achieved using digital signature-based data aggregation is 7–15% higher than that using the existing adaptive traffic segmentation [7] method.

**Figure 8:**
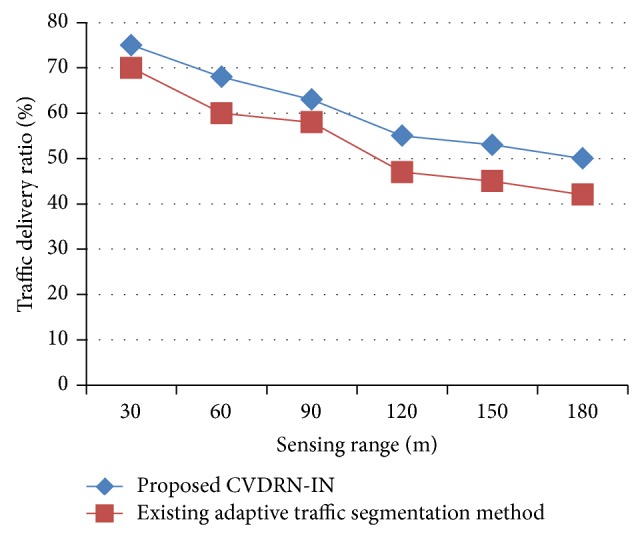
Measure of traffic delivery ratio.

Figure 8 illustrates the traffic delivery ratio. The sensing range of each sensor varies from 30 to 180 meters. Each simulation time was configured for 50 seconds, with each simulation being carried out 4 times on a random topology to normalize the graph. The traffic delivery ratio offered using CVDRN-IN is higher than that using the existing adaptive traffic segmentation [7] method, though with the increasing range of sensors the delivery ratio is reduced. Comparatively, it is higher using CVDRN-IN than with the existing model. The traffic delivery ratio was higher because the enhanced data aggregation is performed by encrypting the MVs destined to the base station and then by checking the validity of the aggregation results based on the digital signature ensuring higher traffic delivery ratio. The variance achieved using CVDRN-IN is 7–10% higher.

Finally, it is being observed that the proposed CVDRN-IN in road network using enhanced data aggregation with intention numeration method identified the movement of vehicle and collects the information about the direction in which it moves based on clustering. The adaptive node clustering clustered the sensor nodes based on the vehicle information collection. A polynomial regression function is presented to identify the cluster head among the set of sensor nodes in the network. Finally, enhanced data aggregation is achieved using the message authentication code based on the movement of vehicles.

## 5. Conclusions

In this work, the intention numeration method based on node energy (IN-node energy) is developed for the identification of vehicles. At first, the sensor nodes in the road network are fixed in an appropriate area. It detects the movement of vehicles and performs the clustering as separate clusters and then computes the number of vehicles covered by every cluster. After the formation of clusters and the vehicle analysis, a polynomial regression function is presented to form the cluster head. A polynomial regression function is then applied to determine the node drain rate strength over the formed cluster. Then the total node energy capacity is estimated by taking the integral of the function over the area. Finally, a conceptual framework is provided for enhanced data aggregation using digital signature model to minimize the amount of data transmitted and avoid redundancy. Experimental evaluation is performed with Dodger's loop data set extracted from UCI repository to estimate the performance of the vehicle movement in the road network. Performance evaluation shows that the proposed CVDRN-IN method provides 10–12% increase in vehicle detection rate in road network.

## Figures and Tables

**Algorithm 1:**
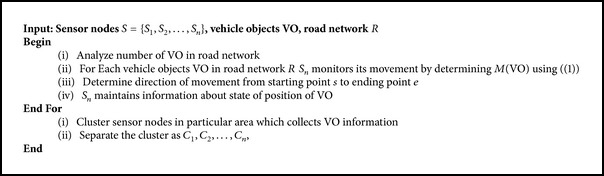
Adaptive node energy clustering.

**Table 1:** Constraints description.

Attributes	Format
Date	MM/DD/YY
Time	(H) H:MM (military time)
Count	Number of cars measured for the previous five minutes
Beginning event time	HH:MM:SS (military)
Ending event time	HH:MM:SS (military)
Away team	—
Game attendance	—
*W*/*L* score	—

## References

[1] Shuai M., Xie K., Xiujun M., Song G. An on-road wireless sensor network approach for urban traffic state monitoring.

[2] Lin Y.-H., Chang S.-Y., Sun H.-M. (2013). CDAMA: concealed data aggregation scheme for multiple applications in wireless sensor networks. *IEEE Transactions on Knowledge and Data Engineering*.

[3] Gupta R., Ramamritham K. (2012). Query planning for continuous aggregation queries over a network of data aggregators. *IEEE Transactions on Knowledge and Data Engineering*.

[4] Sabbineni H., Chakrabarty K. (2010). An energy-efficient data delivery scheme for delay-sensitive traffic in wireless sensor networks. *International Journal of Distributed Sensor Networks*.

[5] Saif A. F. M. S., Prabuwono A. S., Mahayuddin Z. R. (2014). Moving object detection using dynamic motion modelling from UAV aerial images. *The Scientific World Journal*.

[6] Ozdemir S., Xia Y. (2009). *Secure Data Aggregation in Wireless Sensor Networks: A Comprehensive Overview*.

[7] Villas L. A., Horizonte B., Boukerche A., Ramos H. S., de Oliveira H. A. B. F. (2013). DRINA: a lightweight and reliable routing approach for in-network aggregation in wireless sensor networks. *IEEE Transactions on Computer*.

[8] Brahme Y. B., Kulkarni P. S. An implementation of moving vehicle detection, tracking and counting vehicles for traffic surveillance system.

[9] Suhr J. K., Jung H. G. (2013). Sensor fusion-based vacant parking slot detection and tracking. *IEEE Transactions on Intelligent Transportation Systems*.

[10] Hao J. Y., Li C., Kim Z., Xion Z. (2013). Spatio-temporal traffic scene modeling for object motion detection. *IEEE Transactions on Intelligent Transportation Systems*.

[11] Falcone P., Colone F., Lombardo P., Pastina D. WiFi-based passive ISAR for high resolution cross-range profiling of moving targets.

[12] Mithun N. C., Rashid N. U., Rahman S. M. M. (2012). Detection and classification of vehicles from video using multiple time-spatial images. *IEEE Transactions on Intelligent Transportation Systems*.

[13] Cherng S., Fang C.-Y., Chen C.-P., Chen S.-W. (2009). Critical motion detection of nearby moving vehicles in a vision-based driver-assistance system. *IEEE Transactions on Intelligent Transportation Systems*.

[14] Radha Krishna Reddy P., Joshna P., Sireesha G., Thirupathaiah A. Data collection through vehicular sensor networks by using TCDGP. http://arxiv.org/abs/1206.6281.

[15] Umedu T., Suita I., Higashinoz K., Toh C. K. (2009). An intervehicular-communication protocol for distributed detection of dangerous vehicles. *IEEE Transactions on Vehicular Technology*.

[16] Daigle J. N., Sun Y. (2007). *Intelligent wireless sensor network based vehicle detection and classification [Doctoral Dissertation]*.

[17] Dietzel S., Kargl F., Heijenk G., Schaub F. On the potential of generic modeling for VANET data aggregation protocols.

[18] Ozdemir S., Çam H. (2010). Integration of false data detection with data aggregation and confidential transmission in wireless sensor networks. *IEEE/ACM Transactions on Networking*.

[19] Jung W.-S., Lim K.-W., Ko Y.-B., Park S.-J. A hybrid approach for clustering-based data aggregation in wireless sensor networks.

[20] Shih F. Y., Wu Y.-T., Chuang C.-F., Chen J.-L., Lu H.-F., Chang Y.-C. (2007). An intelligent sensor network for object detection, classification and recognition. *Journal of Information Science and Engineering*.

